# Application of Composite Raman Probes in Tumor Diagnosis and Imaging

**DOI:** 10.3390/polym18070843

**Published:** 2026-03-30

**Authors:** Shuting Zou, Yue Wen, Wanneng Li, Huanhuan Sun, Hongyi Yin, Dean Tian, Sidan Tian, Mei Liu, Jun Liu

**Affiliations:** 1 Third Hospital of Shanxi Medical University, Shanxi Bethune Hospital, Shanxi Academy of Medical Sciences, Tongji Shanxi Hospital, Taiyuan 030032, China; zoushuting@sxmu.edu.cn (S.Z.); zenmehaobanzenmeban@sxmu.edu.cn (H.S.); yinhongyi@sxmu.edu.cn (H.Y.); datian@tjh.tjmu.edu.cn (D.T.); 2 Department of Gastroenterology, Tongji Hospital, Tongji Medical College, Huazhong University of Science and Technology, Wuhan 430022, China; wenyue@tjh.tjmu.edu.cn (Y.W.); d202582809@hust.edu.cn (W.L.); 3 National Engineering Research Center for Nanomedicine and Key Laboratory of Molecular Biophysics of Minister of Education, College of Life Science and Technology, Huazhong University of Science and Technology, Wuhan 430074, China; sdtian1616@hust.edu.cn

**Keywords:** composite Raman probes, surface-enhanced Raman scattering (SERS), tumor diagnosis, plasmonic nanostructures, molecular imaging, cancer theranostics

## Abstract

Raman spectroscopy offers unique molecular fingerprinting capability for cancer diagnosis and monitoring, yet its biomedical application is fundamentally limited by weak intrinsic signals and complex biological backgrounds. Composite Raman probes, particularly surface-enhanced Raman scattering (SERS)—based systems, overcome these limitations through synergistic electromagnetic and chemical enhancement combined with functional integration. By engineering plasmonic nanostructures, interfacial electronic states, and molecular architectures, composite Raman probes achieve synergistic electromagnetic and chemical enhancement while incorporating biorecognition units, reporter molecules, and protective coatings to improve stability, specificity, and biocompatibility. In recent years, these probes have evolved from simple signal tags into multifunctional platforms capable of ultrasensitive tumor biomarker detection, high-contrast imaging, surgical guidance, therapy monitoring, and dynamic analysis of the tumor microenvironment (TME). This review systematically summarizes recent advances in composite Raman probes for oncological applications, with an emphasis on material design strategies, enhancement mechanisms, and stimulus-responsive regulation. Representative applications at both molecular and tissue levels are highlighted, including nucleic acid, protein, and exosome detection, as well as in vivo imaging and microenvironmental sensing. Finally, current challenges and future perspectives toward clinical translation are discussed, aiming to provide guidance for the rational design of next-generation Raman probes for precision oncology.

## 1. Introduction

Early diagnosis and precise therapy of malignancies remain major challenges in clinical oncology. Despite rapid advancements in imaging, histopathology and molecular biology, the ability to identify and monitor tumors at early stages with high spatial resolution and minimal invasiveness remains a critical challenge. Among spectroscopic techniques, Raman spectroscopy has attracted increasing attention as a powerful biomedical analytical tool owing to its unique molecular fingerprinting capability, weak interference from water, label-free operation, and potential for real-time monitoring [1,2]. However, the inherently low Raman scattering cross-sections of biological tissues, together with strong background interference, severely limit detection sensitivity in complex biological systems.

Surface-Enhanced Raman Scattering (SERS) offers a powerful solution to these limitations. By combining electromagnetic mechanism (EM) enhancement from metallic nanostructures with chemical mechanism (CM) enhancement arising from molecular adsorption, SERS can amplify Raman signals by several orders of magnitude, enabling highly sensitive molecular detection in complex biological environments [3,4]. However, detection systems relying solely on label-free single-material platforms require direct physical interaction between analytes and metallic nanostructures. This requirement is often constrained by analyte structural properties, thereby rendering species with low metal affinity, such as carbohydrates and lipids, difficult to detect directly. Consequently, such systems frequently fail to meet the comprehensive requirements of tumor theranostics in terms of sensitivity, specificity, and imaging capability. To address these challenges, increasing attention has been devoted to the development of advanced composite Raman probes, achieved by tailoring nanostructure morphology and size or integrating functional units such as Raman reporter molecules, biorecognition elements, and polymeric coatings to improve signal stability, biosafety, and multifunctional integration [5,6,7].

**Figure 1:**
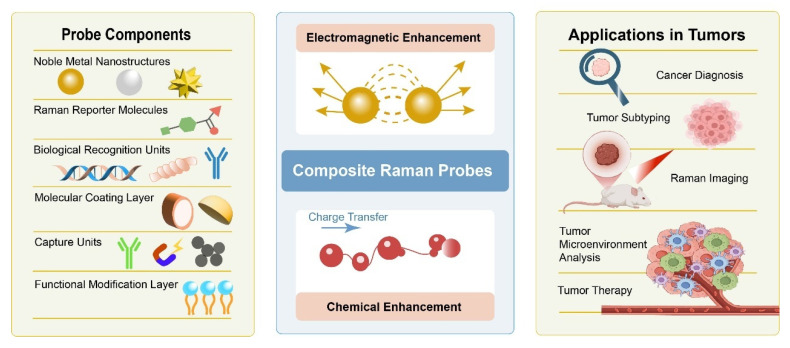
Application of composite Raman probes in tumors.

In oncological applications, composite Raman probes have demonstrated distinct advantages across several critical domains. In the detection of early tumor markers, SERS-based probes enable highly sensitive and specific analysis of circulating tumor cells (CTCs) and exosomes, offering new opportunities for early screening and non-invasive diagnosis [8]. In vivo imaging, composite probes with near-infrared (NIR) enhancement capabilities have been successfully applied to tumor localization and lesion boundary delineation by providing high-contrast visualization of deep tissues [9]. In surgical navigation, SERS probes assist surgeons in intraoperatively distinguishing neoplastic from healthy tissues, thereby improving resection accuracy [10]. Moreover, SERS-based platforms enable real-time monitoring of therapeutic processes, including drug release, photothermal therapy (PTT), and photodynamic therapy (PDT) [11,12,13]. Finally, in TME analysis, stimuli-responsive composite Raman probes allow in situ monitoring of key microenvironmental parameters such as pH, reactive oxygen species (ROS), and metabolites, providing powerful tools for investigating tumor progression [9,14] (Figure 1).

The central design principle of composite Raman probes lies in the synergistic interplay between material structure and interface engineering to enable efficient amplification and stable regulation of Raman scattering signals, thereby satisfying the stringent imaging and analytical requirements of complex biological systems, particularly the TME. Noble metal nanostructures (e.g., gold and silver) continue to serve as the cornerstone of high-sensitivity SERS probes due to their ability to generate highly localized electromagnetic “hotspots” at localized surface plasmon resonance (LSPR) frequencies. Intensely concentrated electric fields formed at nanogaps, sharp tips, or hierarchical geometries can yield Raman enhancement factors (EF) of up to 10^8^, providing the fundamental amplification framework for composite probe construction [4].

Ideal Raman probe architecture should exhibit biocompatibility, reproducibility, and long-term stability under physiological conditions. Addressing the challenges associated with biomedical SERS, which include limited functionality, insufficient targeting selectivity, and susceptibility to interference from complex biological matrices, remains essential for clinical translation. However, single noble metal nanostructures often exhibit limited chemical selectivity, structural stability, and functional scalability. Consequently, the development of composite systems incorporating biomolecules, multicomponent metals, semiconductors, or carbon-based materials has emerged as a prevailing trend. Although semiconductor materials (e.g., TiO_2_, ZnO, MoS_2_) and carbon-based materials (e.g., graphene) can enhance chemical contributions through charge transfer or molecular adsorption [15], such systems typically function as passive detection substrates rather than active probes with targeting and in vivo applicability and are therefore beyond the primary scope of this review. In contrast, integrating Raman reporter molecules, polymer coatings, stimuli-responsive layers, and biorecognition units (e.g., antibodies or aptamers) confers enhanced signal stability, controlled release, targeted accumulation, and microenvironmental responsiveness, thereby substantially improving probe operability and biological adaptability.

As material systems continue to diversify, the design of composite Raman probes has evolved from a singular emphasis on signal amplification toward the development of integrated platforms featuring multidimensional functionality, including synergistic enhancement mechanisms, programmable architectures, controllable biological behaviors, and intelligent microenvironmental responsiveness. Notwithstanding substantial progress, composite Raman probes continue to face several critical challenges, such as limited optical penetration in deep-tissue imaging, uncertainties surrounding in vivo metabolism and long-term biosafety, reproducibility issues during probe fabrication, and barriers to scalable production and clinical translation [16]. Accordingly, this review systematically summarizes and critically analyzes recent advances in composite Raman probes for tumor theranostics, with a particular focus on studies reported predominantly over the past three years.

We first classify the construction strategies of composite Raman probes from a materials design perspective, with a distinct focus on their material engineering and functional integration, rather than merely emphasizing detection applications or platform integration. Taking “material design, enhancement mechanisms, and stimulus-responsive regulation of composite Raman probes” as the core thread, this review systematically elaborates on four major strategies: plasmonic structural engineering, interfacial electronic modulation, molecular structure modification, and dynamic regulation driven by bio/chemical stimuli. In addition, new systems such as stimulus response probes, ratio probes, and self-assembled hotspot probes that were not covered by similar reviews were also added. Next, by integrating these strategies with oncology research, this review covers the full spectrum of applications, ranging from molecular-level biomarker detection to tissue-level tumor imaging, intraoperative navigation, and dynamic TME analysis. This stands in contrast to most existing reviews, which focus heavily on in vitro detection, liquid biopsy, and biomarker quantification, with only fragmentary coverage of tumor imaging, intraoperative navigation, and TME-related analysis Finally, current limitations and future perspectives are discussed to provide conceptual and practical guidance for advancing probe design and accelerating clinical translation.

## 2. Classification and Construction Strategies of Composite Raman Probes

For clarity, a composite Raman probe is defined as an integrated, self-contained, functional Raman-active nanoplatform constructed by synergistically assembling two or more intrinsic functional components, including plasmonic nanostructures, Raman reporter molecules, biorecognition units (antibodies, aptamers), protective layers, targeting moieties, and stimuli-responsive modules. In contrast, standalone external plasmonic detection chips, sensors or substrates function only as passive, fixed, non-targetable signal-enhancing platforms, and therefore do not fall into the category of composite Raman probes. Notably, composite Raman probes can be flexibly coupled with the aforementioned external platforms to facilitate target separation, enrichment, and signal amplification. However, these external chips or sensors lack the self-contained functional integration, active targeting capability, and in situ responsiveness that are essential characteristics of composite Raman probes, so they cannot be defined or regarded as composite Raman probes themselves. In this context, we classify composite Raman probes into the following four categories based on their enhancement mechanisms.

### 2.1. Plasmonic Structural Engineering for Electromagnetic Enhancement

Noble metals (e.g., Au and Ag) remain the most representative plasmonic materials in current SERS applications. Both theoretical analyses and experimental investigations indicate that the SERS enhancement factor scales approximately with the fourth power of the local electric field intensity (|E|^4^). Accordingly, enhancing the local electromagnetic field through rational structural engineering represents one of the most direct and effective strategies to improve SERS sensitivity. Since electromagnetic enhancement primarily originates from the intrinsic properties of plasmonic nanostructures rather than the chemical identity of target molecules, precise modulation of geometric morphology, surface sharpness, and plasmonic coupling modes forms the core design principle of electromagnetically enhanced composite Raman probes.

Structurally, plasmonic engineering strategies for enhancing electromagnetic fields generally follow two major trajectories. The first involves constructing anisotropic single-particle nanostructures with sharp morphological features, such as gold nanostars and nanoflowers. These structures preferentially concentrate surface charges at vertices or tips, giving rise to highly localized, single-particle–dominated hotspots capable of generating pronounced electromagnetic enhancement even under isolated conditions. The second trajectory relies on controlled assembly or template-assisted fabrication to introduce nanoscale gaps that promote plasmonic coupling; within such gap regions, the local enhancement factor can increase by several orders of magnitude [17]. These two approaches exhibit distinct characteristics in terms of hotspot distribution, structural stability, and applicability across different SERS scenarios.

#### 2.1.1. Anisotropic Single-Particle & Multi-Metallic Structures

**Figure 2:**
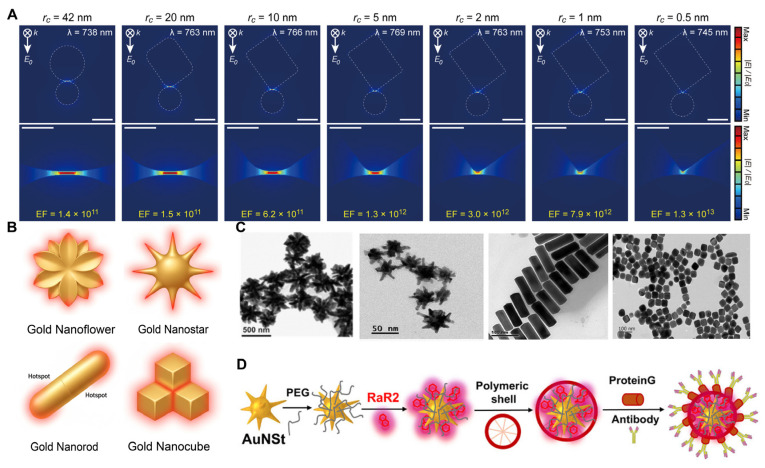
(**A**) The electric field variation near the nanogap region of nanocubes with different curvature radii. Reproduced with permission from [18], American Chemical Society, 2016. (**B**) Schematic illustrations of gold nanoflowers, gold nanostars, gold nanorods, and gold nanocubes, presented sequentially. (**C**) from left to right are the bright field STEM (BF-STEM) image of Au NF, Reproduced with permission from [26], American Chemical Society, 2025.TEM of Au NS. Reproduced from [22], Wiley-VCH GmbH, Weinheim, 2025. TEM of AuNR. Reproduced from [27], Tsinghua University Press, 2025. TEM image of AgNC, Reproduced from [25], American Chemical Society, 2025. (**D**) A typical composite Raman probe prepared based on anisotropic metal nanoparticles. Reproduced from [22], Wiley-VCH GmbH, Weinheim, 2025.

Anisotropic single-particle nanostructures represent one of the most extensively explored strategies in plasmonic structural engineering. As shown in Figure 2A, In the gold nanocube (AuNC) nanosphere dimer, as the curvature radius of the nanocube gradually decreases, that is, as the cube edges become sharper, the degree of localization of the induced electric field around the vertices significantly increases, and the SERS enhancement factor can even reach the order of 10^13^ [18]. In addition, by comparing the SERS spectra and enhancement factors of Ag nanocubes and nanospheres modified with Raman probes deposited on different substrates, it was found that hotspots only occur when nanoparticles with sharp angles come into contact with metal surfaces [19]. All of the above indicate that compared with spherical nanoparticles, metallic nanostructures featuring high-curvature tips, edges, or reduced symmetry are more prone to localized charge accumulation under external optical excitation, thereby generating intense, spatially confined electric fields at vertices or protrusions. Typical representatives include old nanoflower, gold nanostar, gold nanorod (AuNR), and AuNC. Figure 2B and Figure 2C, respectively, show schematic diagrams and Transmission Electron Microscope (TEM) images of classical anisotropic nanoparticles. A key advantage of these structures lies in their ability to achieve substantial SERS enhancement primarily relying on single-particle tip-induced effects, even in the absence of strong interparticle coupling, rendering them particularly suitable for the construction of probe-type SERS nanotags.

In practical applications, anisotropic plasmonic structures are typically functionalized with Raman reporter molecules, followed by bioconjugation with antibodies, aptamers, or peptides to integrate signal amplification with molecular recognition. For example, Wang et al. fabricated silver nanoflower-based immuno-SERS probes by covalently coupling Raman reporters and enzyme-specific antibodies, achieving high SERS activity with effective immune recognition [20]. Zheng et al. constructed bimetallic Ag-Au nanoflowers with intrinsic self-reporting properties, in which abundant anisotropic tips served as plasmonic hotspots while embedded reporters provided stable SERS responses [21]. Similarly, Piñeiro et al. (Figure 2D) [22] and Ngo et al. [23] separately employed gold nanostars as anisotropic plasmonic substrates and developed composite SERS probes by functionalizing nanostar surfaces with different Raman reporters and targeting antibodies, yielding probe platforms with analogous structures but distinct functionalities. Lan et al. prepared gold nanorod-based immuno-SERS signal probes incorporating Raman reporters and capture antibodies, enabling potent signal amplification and specific molecular recognition [24]. Furthermore, Chen et al. designed composite probes consisting of polystyrene microspheres, gold nanocubes, and CD9 antibodies for the specific SERS-based identification of exosomes [25]. Collectively, these works validate the applicability of anisotropic single-particle plasmonic architectures in probe-type SERS platforms for bioanalytical.

Although anisotropic single-particle systems exhibit remarkable advantages, including structural simplicity, signal stability, and facile surface functionalization, they possess distinct inherent limitations. Direct contact between the plasmonic metal and the surrounding chemical environment readily induces particle aggregation and uncontrolled release, while the hotspots are confined to a small number of tip sites [28]. These two critical issues directly result in inadequate stability and hotspot density of the nanoprobes, further restricting their applicability in practical scenarios such as large-area sensing and ultra-trace detection.

To mitigate these limitations—specifically, to block direct contact between plasmonic metals and the external chemical environment, alleviate particle aggregation, and resolve derived drawbacks including poor biocompatibility and uncontrolled ion release—researchers have introduced inert shell encapsulation strategies into the design of composite Raman probes, offering an effective avenue to overcome these bottlenecks. The feasibility and efficacy of this strategy have been validated in numerous studies: for instance, Kirche et al. [6] and Bagheri et al. [29] encapsulated plasmonic nanostructures with silica shells, positioning Raman reporters either within the core–shell interspace or on the outer shell to enhance chemical stability while maintaining SERS activity; Song et al. [30] and Guo et al. [31] employed polydopamine coatings to encapsulate Raman reporters, improving probe stability and biocompatibility. Cheng et al. utilized poly(styrene-maleic acid) (PSMA) as a stabilizing layer to regulate nanoparticle dispersion and surface functionality [32]; additionally, Zhang et al. introduced copper sulfide shells to prevent the detachment or leakage of Raman reporters under in vivo conditions [33]. These shell-isolated plasmonic architectures effectively preserve SERS signal integrity while substantially improving chemical robustness and long-term performance in nanoprobe-based applications.

Despite the above improvements offered by inert shell passivation, certain structural constraints persist in representative core–shell designs, as exemplified by the work of Lei et al. [34]. These authors fabricated silver-coated AuNRs functionalized with DNA aptamer recognition moieties to develop SERS probes with enhanced plasmonic activity. In this architecture, Raman reporters anchored on the outer surface of the silver shell only undergo relatively weak near-field enhancement. While interparticle aggregation can generate additional interparticle hotspots, this enhancement mode remains largely uncontrolled. Coupled with the intrinsically limited hotspot density of anisotropic single-particle systems, these shortcomings emphasize the demand for more rational structural design strategies—specifically, gap engineering and spatially defined hotspot construction—in next-generation high-performance composite Raman probes.

#### 2.1.2. Plasmonic Coupling and Gap-Mode Hotspot Strategies

**Figure 3:**
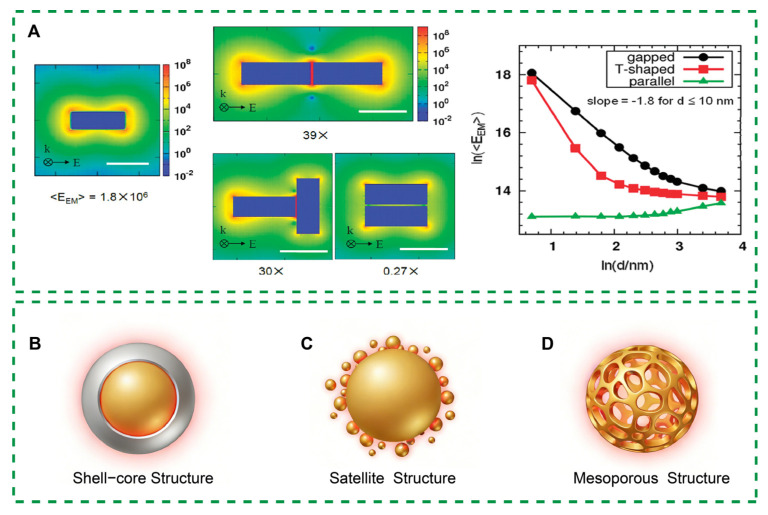
(**A**) The influence of particle gaps on the electromagnetic contribution of SERS enhancement. Reproduced with permission from [38], American Chemical Society, 2011. (**B**–**D**) Pattern diagram of three coupling strategies to enhance hotspots.

To address the intrinsic limitation of restricted hotspot numbers in anisotropic single-particle systems, plasmonic coupling-based enhancement strategies have been widely developed. The central concept underlying this approach is the introduction of well-defined nanoscale gaps between adjacent plasmonic units, which induces strong near-field coupling and gives rise to gap-mode hotspots. Compared with single-particle tip-induced hotspots, gap-mode electromagnetic fields are more strongly confined and can result in local enhancement factors increasing by several orders of magnitude when the gap distance is reduced to the nanometer or sub-nanometer scale [35,36,37]. As illustrated in Figure 3A, Electromagnetic Enhancement Factor (E_EM_) with different rod-like dimer structures is affected by the size of the gaps. For head-to-head and vertical dimers, narrow gaps favor the generation of high enhancement factors. In contrast, parallel rod-shaped dimers exhibit no prominent hotspots between particles, arising from destructive interference of the induced symmetric dipoles within each constituent particle; notably, such ideal symmetric alignment is rarely encountered in practical systems [38].

Therefore, to achieve ideally controllable gap-mode hotspots, three archetypal structural platforms have been established: core–shell structures, satellite assemblies, and mesoporous nanostructures. Before the widespread adoption of these mainstream configurations, physical immobilization strategies using templated scaffolds have served as a foundational proof-of-concept route toward regulated nanogap formation. (Figure 3B–D).

**Figure 4:**
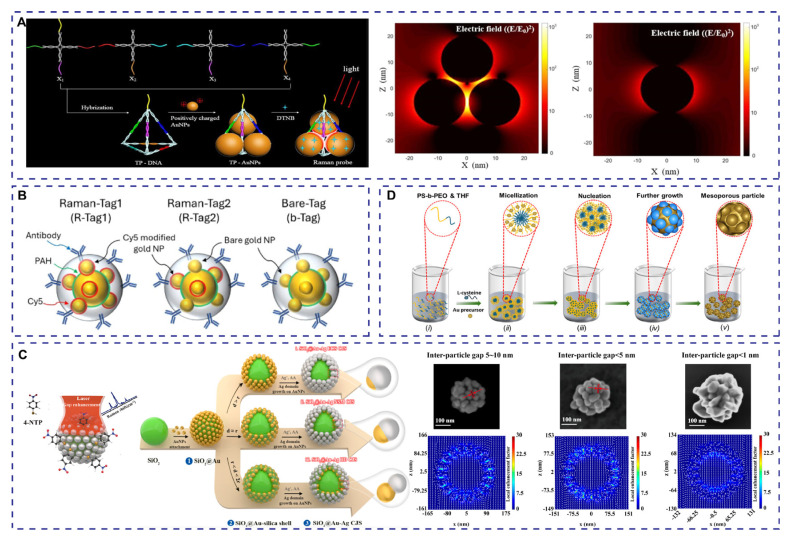
(**A**) Scheme for assembling gold nanoparticle Raman probes through triangular cone DNA assembly. Reproduced with permission from [39], American Chemical Society, 2019. (**B**) Scheme for constructing SERS probes based on gold core satellite nanostructures. Reproduced from [50], American Chemical Society, 2025. (**C**) SERS probe based on tunable CJS structure and its electromagnetic field variation. Reproduced with permission from [54] American Chemical Society, 2024. (**D**) Synthesis of spherical mesoporous gold nanoparticles. Reproduced from [55], Wiley-VCH GmbH, Weinheim, 2025.

As a representative physically templated approach, DNA-mediated nanoparticle assembly offers a facile means to fix interparticle distances and preliminarily realize controllable plasmonic coupling. Zhang et al. constructed gold nanoparticle (Au NP) assemblies via electrostatic attraction between negatively charged DNA tetrahedral scaffolds and positively charged AuNPs, enabling homogeneous interparticle gap formation and site-specific functionalization of recognition moieties, as depicted in Figure 4A [39]. Similarly, Wu et al. [40] and Sharma et al. [41] utilized rectangular DNA origami templates to precisely regulate interparticle spacing between adjacent gold nanostructures, resulting in reproducible and controllable SERS signal amplification. Both strategies rely on the precise regulation of plasmonic coupling gaps to construct of SERS signal-amplifying probes.

Bimetallic core–shell architectures represent one of the most widely adopted gap-mode strategies in composite Raman probe design. In these systems, Raman reporter molecules are strategically positioned within the nanoscale core–shell interspace, where the confined gap acts as a robust internal hotspot. This architecture effectively insulates reporters from the external environment while retaining strong electromagnetic enhancement. Representative work by Fu et al. [42], Zhang et al. [43], Liu et al. [44], Shah et al. [45], Wen et al. [46], Xia et al. [47], and Fan et al. [48] demonstrated that Au@Ag or Ag@Au core–shell nanostructures with gap-embedded reporters exhibit high signal reproducibility and improved long-term stability and can be readily functionalized with targeting ligands for specific bioanalytical applications. In addition, Li et al. reported AgAu@Ag triangular nanonetwork alloy structures [49], further extending gap-mode enhancement through multimetallic coupling and interconnected geometries.

Satellite-type plasmonic architectures represent another important category of gap-mode enhancement systems, combining multi-particle coupling with multifunctional probe integration. By anchoring multiple satellite nanoparticles onto a central core, dense interparticle nanogaps can be generated, producing concentrated electromagnetic hotspot regions. For instance, Chang et al. fabricated 50 nm Au core-satellite nanostructures with 20 nm Au satellite NPs (Figure 4B), then integrated three Raman reporters and antibodies to yield targeted probes with stable SERS responses and efficient photothermal conversion [50]. Sun et al. further developed a gold yolk-shell-satellite architecture by growing small gold nanoparticles in situ on hollow gold nanocages, generating dense plasmonic hotspots while enabling directional antibody immobilization via bifunctional linkers [51].

Beyond fixed satellite configurations, modulation of satellite size, spacing, and loading density has also been employed to regulate plasmonic coupling strength. In this context, Mellor et al. directed ultrasmall Au NPs to self-assemble into stable nanoclusters within a polymer matrix, achieving tunable cluster dimensions and interparticle gaps as well as synergistic SERS and near-infrared photothermal enhancement by varying the Au/polymer ratio [52]. Similarly, Liu et al. engineered Au-Ag composite nanoplates with evolution from 2D island-like morphologies, to 3D-on-2D configurations, and finally to fully 3D architectures; precise regulation of nanogap dimensions and surface topography allowed optimization of plasmonic coupling [53]. Furthermore, Hao et al. revealed that prominent SERS enhancement arises not only from interparticle coupling between satellite units but also from intraparticle coupling within individual satellites. As shown in Figure 4C, they developed a tunable core-Janus-satellite (CJS) structure using silicon spheres as the core and Au-Ag Janus domains as satellite components. By engineering the heterojunction morphologies of these Janus satellites, they finely modulated internal neck gaps to maximize electromagnetic field enhancement, moving beyond conventional strategies that only adjust satellite size or loading density to regulate SERS performance [54].

Mesoporous plasmonic nanostructures offer a complementary gap-associated enhancement route that combines molecular confinement with plasmonic amplification. In such systems, Raman reporters are immobilized within nanoscale pores, mitigating signal fluctuations from Brownian motion while providing abundant loading sites. Bashir et al. reported mesoporous gold nanospheres with tunable pore dimensions and high surface area; their synthetic route is illustrated in Figure 4D. The mesopores enable high-capacity loading of Raman reporters and antibodies for robust SERS nanotag fabrication [55]. Hwang et al. grew Raman-labeled Ag nanogap shells inside the confined pores of mesoporous silica nanoparticles, markedly boosting SERS signal intensity and stability [56]. Likewise, Ahmed et al. developed mesoporous gold nanotags loaded with multiple Raman reporters and functionalized with cognate antibodies, realizing efficient hotspot formation and multiplexed SERS detection [57].

It is worth noting that although the targeting function of antibodies renders them an indispensable component of composite Raman probes, their poor stability and high preparation cost have spurred the development and application of molecularly imprinted polymers (MIPs). Regarded as stable, cost-effective, and rationally designable “artificial antibodies”, MIPs serve as an ideal complement or alternative to traditional biological antibodies in tumor detection applications [58]. The integration of MIPs with plasmonic nanomaterials has further offered novel strategies for gap-mode hotspot regulation. For instance, Chen et al. utilized MIP hydrogels to encapsulate silver nanoparticles for the detection of bovine serum albumin (BSA) [59]. Guo et al. demonstrated that coating an ultra-thin MIP shell (typically <10 nm) on the surface of Ag or Au nanoparticles can form core–shell gaps. When target molecules enter the MIP layer, changes in their dielectric properties modulate the local electromagnetic field distribution, generating the so-called “virtual hotspots” or “gating effect”, thereby enhancing Raman signals. This provides a new direction for the intelligent and specific regulation of gap-mode hotspots [60].

Overall, gap-mode plasmonic coupling strategies provide enhanced electromagnetic amplification and improved signal robustness compared with single-particle systems, making them particularly suitable for high-sensitivity bioanalysis. Nevertheless, their performance remains closely associated with nanoscale fabrication precision and structural uniformity, and further optimization is required to balance enhancement efficiency, probe stability, and scalability. Consequently, integrating gap-engineered plasmonic probes with magnetic bead–based separation [47,57,61,62,63,64], MIP [58,65,66,67,68], composite substrates or microfluidic platforms [15,24,49,56,69,70,71,72] to construct sandwich-type immunoassay systems has emerged as a prevailing strategy for simultaneously amplifying SERS signals and improving target enrichment efficiency.

### 2.2. Interfacial Electronic Modulation for Chemical Enhancement

In contrast to EM-dominated strategies that rely primarily on nanostructure-induced field confinement, CM-oriented approaches emphasize interfacial electronic interactions between the substrate and probe molecules. This mechanism posits that charge transfer (CT) at the substrate-molecule interface alters the molecular electron density distribution, thereby enhancing molecular polarizability and increasing the Raman scattering cross-section. Theoretically, the signal amplification achievable through CM is estimated to reach up to 10^3^ [73]. Interfacial electronic modulation strategies aim to enhance Raman signals by regulating CT states and interfacial charge distribution, offering enhancement pathways that extend beyond localized plasmonic “hotspots” and thereby improving probe performance in complex biological systems.

**Figure 5:**
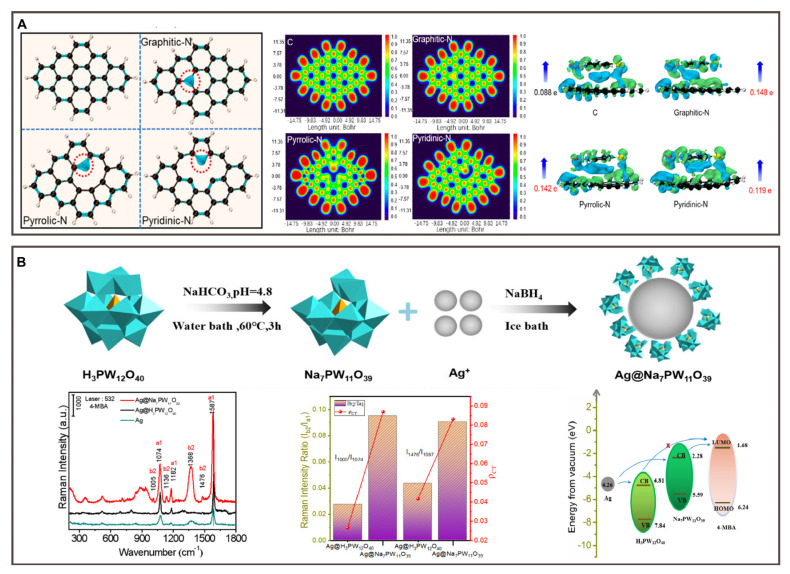
(**A**) Isosurface maps of valence electron density, color filled maps of the Electron Localization Function (ELF) diagram and the charge difference for NCD NCs. The red circle represents the position of N-atom in different structures with N-atom doping. Arrows and numerical values indicate the direction and quantity of charge transfer, respectively. Reproduced with permission from [75], American Chemical Society, 2023. (**B**) Schematic illustration of the synthesis of Ag@ Na_7_PW_11_O_39_ and its CT mechanism. Reproduced with permission from [76], American Chemical Society, 2025.

Wang et al. reported an “electron convergence-enhanced” Raman probe by encapsulating a Raman reporter-functionalized gold shell with an electroactive liposome membrane derived from *Shewanella oneidensis*. Electron injection mediated by c-type cytochromes within the membrane modulated interfacial charge-transfer behavior at the gold shell-reporter interface, enabling CM-dominated SERS enhancement with improved signal stability and reduced reliance on geometric hotspot engineering [74]. Lin et al. developed novel amorphous nitrogen-doped carbon (NDC) nanocages (NCs), in which the surface electronic structure was effectively regulated by specific nitrogen configurations. Figure 5A shows that after N-atom doping, C-based nanomaterials exhibit high electron Density of States (DOS), narrow bandgap, and strong electron delocalization, improving charge transfer efficiency and promoting vibrational coupling in the substrate molecule system [75]. Building upon this concept, Zhao et al. subsequently constructed composite SERS substrates by integrating silver nanoparticles with defect-rich polyoxometalates (POMs), exploiting defect-engineered electronic structures to regulate interfacial charge-transfer pathways and achieve synergistic enhancement. In particular, lacunary POMs (Na_7_PW_11_O_39_) introduce modified Highest Occupied Molecular Orbital—Lowest Unoccupied Molecular Orbital (HOMO–LUMO) energy levels compared with their fully coordinated counterparts (H_3_PW_12_O_40_), thereby facilitating more favorable CT processes under resonant excitation, as schematically illustrated in Figure 5B. Under 532 nm illumination, efficient electronic coupling among Ag, Na_7_PW_11_O_39_, and the Raman probe molecule enabled pronounced chemical enhancement, whereas such CT pathways were largely suppressed in the Ag@H_3_PW_12_O_40_ system. This defect-induced electronic synergy underlies the superior SERS performance observed for Ag@ Na_7_PW_11_O_39_, highlighting the critical role of energy-level engineering in CM-dominated SERS systems [76].

Overall, the contribution of CM to SERS remains complex and multifaceted. Existing studies suggest that semiconductor materials, including graphene, transition metal dichalcogenides (TMDs), transition metal oxides, metal–organic frameworks (MOFs), and conjugated molecules, predominantly contribute to SERS through CT-induced enhancement mechanisms. However, most semiconductor-based systems are implemented as SERS substrates or chip-based platforms, rather than as probe-type architectures suitable for in vivo biomedical applications, and therefore fall outside the primary scope of this review [77].

### 2.3. Molecular Electronic Structure Modulation for Intrinsic Scattering Enhancement

Conventional SERS systems predominantly rely on electromagnetic (EM) enhancement derived from plasmonic coupling and gap-mode hotspots, which delivers remarkable signal amplification but can be constrained by issues including photobleaching of reporters, matrix interference, and structural instability of plasmonic nanostructures in complex physiological environments. To address these limitations and complement EM-dominated enhancement, a fundamentally distinct strategy has emerged that focuses on boosting the intrinsic Raman scattering cross-section of target molecules by modulating their electronic structure and polarizability. This approach strengthens the inherent scattering response of the Raman-active species itself, rather than relying solely on external plasmonic field confinement, thereby offering a promising route to robust, background-suppressed, and high-resolution bioanalytical performance.

**Figure 6:**
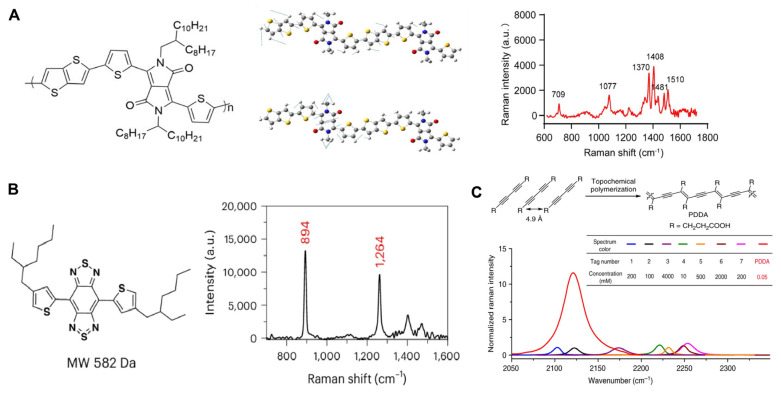
(**A**) The structure, vibrational mode diagram, and solid-state Raman spectrum of D-A conjugated polymer DPPT-TT (785 nm, 0.4225 mW, 1 s exposure time, 5 × objective). Reproduced with permission from [78], American Chemical Society, 2025. (**B**) The Structure and Solid State Raman Spectroscopy of BBT (830 nm, 0.06.1 mW, 5 s exposure time, 20 × objective). Reproduced from [79], Springer Nature, 2025. (**C**) Synthesis of PDDA and Comparison of Raman Intensity with multiple existing Raman probes. Reproduced from [82], Springer Nature, 2020.

The enhancement of a molecule’s intrinsic Raman scattering cross-section is fundamentally governed by the modulation of its electronic structure and polarizability. Qiu et al. demonstrated that constructing donor-acceptor (D-A) conjugated systems (Figure 6A) promotes intramolecular CT and electron delocalization, thereby amplifying the Raman scattering cross-section and markedly enhancing the intrinsic molecular Raman response [78]. They further integrated targeting aptamers and short-half-life radionuclides, enabling the development of multimodal probes for Raman imaging, positron emission tomography (PET) imaging, and PTT. Li et al. developed a self-stacking small-molecule Raman probe (BBT) based on their previously proposed stacking-induced charge-transfer-enhanced Raman scattering (SICTERS) strategy (Figure 6B) [79]. Through ligand functionalization and layer-by-layer encapsulation with chitosan/sodium alginate composite coatings, the probe was endowed with oral administrability and targeted release capability [80].

Beyond conjugated CT systems, bioorthogonal Raman probes bearing isolated vibrational reporters represent another versatile route to intrinsic signal enhancement, particularly for biological imaging applications. Probes functionalized with distinctive vibrational tags, including isotopic labels (C–D), azides, and triple bonds (C≡C, and C≡N), exhibit compelling merits for multiplexed, background-free bioimaging, owing to their characteristic vibrational fingerprints in the Raman-silent window (1800–2800 cm^−1^) and excellent photostability [81]. Accordingly, the integration of intrinsically Raman-active molecules into composite probe architectures has become an increasingly prominent trend.

Tian et al. reported a water-soluble and biocompatible polydiacetylene (PDDA) material exhibiting intrinsically strong Raman activity. As summarized in Figure 6C, systematic comparison with representative state-of-the-art Raman reporters validated its superior signal output characteristics. Derivatives of PDDA were further engineered for stimulated Raman scattering (SRS) imaging, enabling subcellular organelle visualization in HeLa cells [82]. Bao et al. developed a diacetylene-functionalized β-ketoenamine covalent organic framework (BDDA COFs), enabling in vivo imaging of bone microcracks [83]. Although systematic validation of such intrinsic Raman signal-enhanced composite probes in tumor theranostics remains limited, their high signal intensity, excellent photostability, and superior spatial resolution indicate significant potential for expanding biological Raman imaging and analysis applications.

### 2.4. Dynamic Regulation Strategies Driven by Bio/Chemical Stimuli

**Figure 7:**
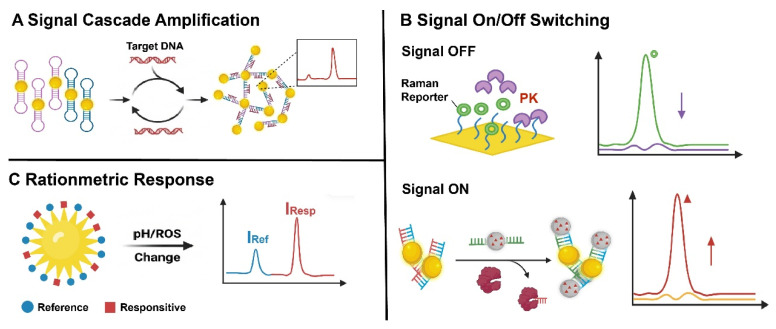
Schematic diagram of SERS probe dynamic regulation strategy: (**A**) Signal Cascade Amplification. (**B**) Signal On/Off Switching. The green circle represents the labeled “Raman reporter” and the purple descending arrow represents the “Signal OFF” after competition. The red triangle represents the Raman signal source on the substance or nanoparticle and the red arrow represents the “Signal ON” generated by the interaction or aggregation of substances. (**C**) Ratiometric Response.

Dynamic regulation strategies triggered by bio/chemical reactions or stimuli have emerged as an important paradigm in the development of Raman probes. These strategies primarily leverage specific biomarkers, metabolic processes, or physicochemical stimuli (pH, ions, enzymes, redox conditions, etc.) to transition the probe’s Raman signal from “static” to “tunable”, “activatable”, or “switchable”, thereby enhancing detection specificity, reducing background interference, and adapting to complex physiological microenvironments. Figure 7 shows three dynamic control strategies, which will be introduced below.

#### 2.4.1. Signal Cascade Amplification

Signal cascade amplification strategies enhance probe sensitivity by coupling two or more amplification processes into a hierarchical “recognition-amplification-re-amplification” scheme, thereby enabling efficient conversion of weak molecular recognition events into robust Raman outputs. Such strategies are particularly well suited for the detection of low-abundance nucleic acids or biomarkers in complex biological environments.

**Figure 8:**
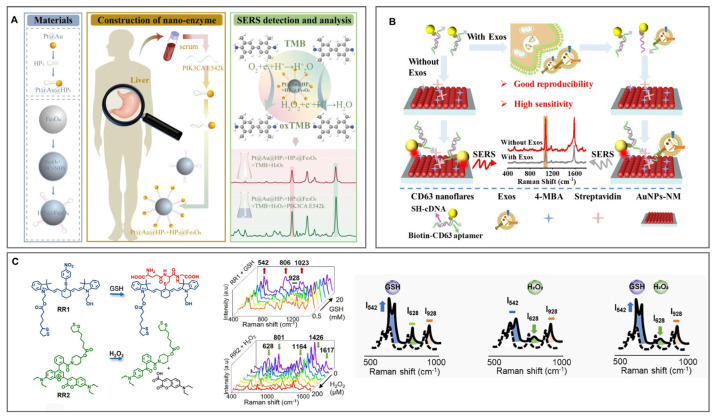
(**A**) Process of target ctDNA-triggered CHA cycle, whose product activates nanozyme to catalyze TMB into ox-TMB with intense Raman signals. Reproduced from [84], Dove Medical Press Ltd., 2025. (**B**) CD63 protein on extracellular vesicle surfaces competes with the biotin-CD63 complex, disrupting the hotspot between AuNPs and CD63 nanoflares and causing a marked decrease in the SERS signal. Reproduced with permission from [91], American Chemical Society, 2025. (**C**) Ratiometric response of the dual-reporter SERS probe toward redox-related metabolites. Reproduced from [101], Wiley-VCH GmbH, Weinheim, 2025.

By combining DNA based amplification reactions with plasma signal transduction, various cascaded amplification SERS platforms have been developed. For example, Wang et al. constructed a multi-level amplification system, in which the target circulating tumor DNA (ctDNA) initiates the catalytic hairpin assembly (CHA) cycle as the primary amplification step, and then the CHA product activates the nanoenzyme to catalyze the conversion of 3,3′, 5,5′—tetramethylbenzidine (TMB) to oxidized TMB for nanoenzyme mediated secondary amplification, thereby generating a strong Raman signal, as shown in Figure 8A [84]. He et al. integrated DNAzymes and hairpin probes onto gold nanoparticle surfaces, enabling target microRNA (miRNA) to trigger CRISPR/Cas13a trans-cleavage and hybridization reactions, which collectively realized SERS signal transduction with cascade amplification [85]. Similarly, Man et al. employed gold nanotetrapods in combination with dual-catalyst hairpin assembly (DCHA), where output DNA strands guided the formation of gold nanotetrapod nanonetworks. Raman reporters incorporated into the assembled networks benefited from hotspot aggregation, resulting in markedly enhanced SERS signals [86].

Beyond conventional hairpin-based amplification, spatial organization and polyvalent capture effects have been exploited to further boost signal output. Liu et al. immobilized Raman reporter-labeled DNA hairpins on the side faces of DNA cubes and anchored them onto gold hexagonal plate substrates. Target-triggered primer exchange reactions (PER) enabled highly specific molecular recognition, while the spatial confinement, polyvalent capture effect, and proximity effect of the DNA cubes dramatically increased the local concentration and fixation efficiency of signal molecules near plasmonic hotspots [87]. Liang et al. designed a cascade amplification system based on Mg^2+^-mediated enzyme-free cleavage and “hand-in-hand” DNA nanostructure cycling, in which target ctDNA induced the generation of abundant signal DNA strands. These strands subsequently directed the self-assembly of gold nanoparticles into plasmonic networks with dense hotspots, effectively transforming weak chemical recognition events into ultrastrong SERS signals [88].

More sophisticated cascade strategies have incorporated logic-gated recognition and enzyme-responsive aggregation to achieve higher specificity and multifunctionality. Xia et al. developed a logic-gated SERS nanoplatform in which gold nanoparticles were functionalized with two hairpin probes responsive to both enzymatic cleavage and target miRNA activation. The simultaneous presence of both biomarkers initiated spatially confined CHA, driving gold nanoparticles to self-assemble into three-dimensional plasmonic networks with abundant hotspots and enabling concurrent photothermal conversion [89]. In a related approach, Swati Tanwar et al. introduced enzyme-responsive peptides onto gold nanocube surfaces, where enzyme-triggered intramolecular condensation reactions induced intracellular aggregation of nanoprobes. This aggregation synchronously produced plasmonic coupling-induced dark-field scattering changes and switchable Raman reporter signals [90].

Overall, recent studies increasingly integrate CHA, hybridization chain reaction (HCR), CRISPR/Cas trans-cleavage, nanozyme catalysis, and plasmonic self-assembly into unified cascade amplification frameworks. By hierarchically coupling molecular recognition with structural reconfiguration and hotspot generation, these strategies enable to achieve highly efficient conversion of chemical recognition events into observable SERS.

#### 2.4.2. Signal on/off Switching

Signal on/off switching strategies enable dynamic regulation of Raman outputs by selectively activating or suppressing probe signals in response to specific biological or chemical stimuli. Depending on the direction of signal variation, these approaches can be broadly classified into “signal-off” and “signal-on” modes.

The “signal-off” mode is widely adopted and typically relies on target-triggered probe dissociation, reporter release, or structural shielding that attenuates Raman enhancement. For instance, Figure 8B schematically illustrates the gold nanoparticle membrane (AuNPs NM) constructed by Diao et al. as a SERS substrate, together with CD63 nanoflares and gold nanoparticle probes modified with their complementary strands, which generate a strong initial SERS signal. Upon introduction of the target, competitive binding between the adaptor and the target induces probe dissociation from the substrate, leading to a pronounced decrease in the SERS signal [91]. Chen et al. constructed a plasmonic-covalent organic framework heterostructure (C@P@A) integrated with a HCR system. Target gene recognition drove hairpin DNA assembly into double-stranded DNA, dynamically modulating the distance between fluorophores and the C@P@A surface and thereby achieving a synergistic “SERS signal turn-off/fluorescence signal turn-on” response [92]. Qiu et al. designed hyaluronic acid–encapsulated silver nanoparticle probes in which hyaluronidase specifically hydrolyzed the responsive surface layer, inducing structural changes and enhanced shielding effects that led to substantial attenuation of SERS signals [93]. In addition, Wang et al. [94] and Yu et al. [95] demonstrated signal-off detection strategies using glass capillaries and glass nanopipettes as core carriers, where aptamer-target protein binding-induced reporter dissociation and protease-triggered reporter release enabled selective detection of target proteins and protease activity, respectively.

In contrast, the “signal-on” mode achieves Raman signal enhancement through target-induced probe assembly, hotspot formation, or enrichment of signal molecules in plasmonic regions. Ye et al. developed a signal-activation strategy based on a core–shell nanostructure and the biotin-streptavidin recognition system. In this design, target DNA activated CRISPR/Cas12a trans-cleavage of biotin-modified spherical nucleic acids (biotin-SNAs), releasing biotin-DNA that competitively bound to magnetic beads and reduced bead-mediated capture of SNAs, ultimately leading to SERS signal activation with improved sensitivity [96]. Chen et al. combined silver nanoparticles with DNA probes complementary to miR-21, where hybridization between the probe and target miRNA generated DNA–RNA duplexes that translated into amplifiable SERS signals through LSPR effects [97]. Zhuang et al. reported a dual-component system consisting of Au nanobipyramid@Ag shell nanoprobes carrying Raman reporters and single-stranded DNA2, together with a graphene-like gold nanohexagon array substrate modified with aptamers and single-stranded DNA1. Target protein recognition triggered aptamer-mediated DNA strand displacement, anchoring the nanoprobes onto the substrate and inducing substantial SERS signal enhancement [98]. Yao et al. synthesized cobalt-tannic acid (TA) nanoparticles loaded with silver nanoparticles and functionalized them with 3-mercaptophenylboronic acid (3-MPBA). By exploiting the Ag-Co bimetallic active centers to enhance nanozyme activity, they achieved chiral recognition and Raman signal modulation of amino acids through specific esterification reactions [99]. Similarly, Zhu et al. modified gold nanoparticles with anti-6-FAM antibodies and Raman reporters, enabling selective binding to FAM-labeled DNA fragments generated by CRISPR/Cas13a cleavage and producing quantifiable SERS signals [100].

Overall, such probes are typically based on structural reconstruction, enzyme digestion, competitive binding, and other methods to achieve “on-off” switching of Raman detection signals, which can significantly suppress background signals and enhance detection specificity.

#### 2.4.3. Ratiometric Response

Ratiometric response strategies enable quantitative Raman analysis by simultaneously monitoring two or more spectrally distinct Raman signals and using their intensity ratio as the analytical output.

Representative ratiometric SERS probes have been constructed by integrating multiple Raman reporters with distinct response characteristics into a single nanoplatform. For example, Yin et al. employed gold nanostars as carriers and modified their surfaces with two Raman reporters possessing different molecular backbones that respond selectively to distinct redox-related metabolites, as shown in Figure 8C. By optimizing the reporter ratio to avoid spectral interference, a ratiometric SERS probe capable of reliable redox sensing was achieved [101]. Koyel Dey et al. covalently functionalized silver nanoparticles with the pH-sensitive Raman molecule 4-mercaptobenzoic acid (4-MBA), forming stable complexes that enabled ratiometric Raman readout of solution pH variations [102]. Cheng et al. further expanded this concept by using bimetallic Ag-Au nanoflower alloys as SERS substrates and incorporating Raman reporters located in the Raman-silent region as dual SERS tags, thereby constructing a ratiometric-responsive probe with minimized background interference [103].

By introducing internal reference signals, these approaches effectively compensate for fluctuations arising from probe concentration, laser power, optical path length, and environmental heterogeneity, thereby significantly improving the accuracy and robustness of Raman-based quantification in complex biological systems.

## 3. Applications of Composite Raman Probes in Oncology

**Figure 9:**
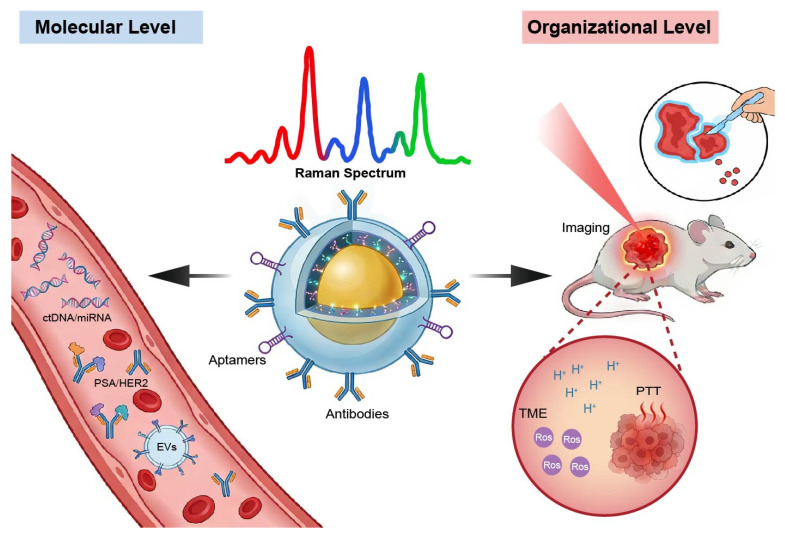
Schematic diagram of the application of composite Raman probes in tumors.

Tumor initiation and progression are governed by a series of highly complex and dynamic processes, including genetic mutations, metabolic reprogramming, and continuous remodeling of the TME. Precisely capturing such multidimensional information in a timely and dynamic manner is central to the development of precision theranostics [104]. In this context, composite Raman probes have emerged as powerful analytical tools, enabling the translation of Raman spectroscopy into clinically relevant diagnostic and therapeutic applications. As shown in Figure 9, driven by advances in structural engineering and functional integration, composite Raman probes have evolved from simple signal labels into multifunctional platforms capable of molecular diagnosis, tumor typing, microenvironmental monitoring, and therapy guidance. According to detection scale and application scenarios, their oncological applications can be broadly classified into molecular-level detection and tissue-level detection.

### 3.1. Molecular-Level Tumor Detection

Molecular-level tumor detection focuses on tumor-associated biomolecular markers, including nucleic acids, proteins, metabolites, and extracellular vesicles (EVs). Conventional analytical techniques for these biomarkers are often labor-intensive, time-consuming, and reliant on expert interpretation. Ideal cancer screening demands short detection times, high precision, and high sensitivity for low-concentration samples [105]. In contrast, composite Raman probes offer rapid readout, ultrahigh sensitivity, and strong resistance to background interference, making them particularly suitable for detecting low-abundance targets in complex biological matrices. As a result, bioanalysis remains the most mature and widely applied oncological application of composite Raman probes. Table 1 summarizes the key parameters of composite Raman probe-based detection for different tumor-associated biomolecular markers, including target type, disease type, target analyte, detection limit (LOD), EF, and recovery rate. This comparative analysis highlights the advantages and limitations of various composite Raman probes, providing valuable insights for the design of next-generation tumor detection platforms.

#### 3.1.1. Nucleic Acid Biomarkers

Tumor-associated nucleic acid biomarkers, such as miRNAs, ctDNA, gene mutations, and viral gene sequences, reflect early molecular abnormalities during tumorigenesis and are therefore highly valuable for precision diagnosis and molecular typing. However, their extremely low abundance and susceptibility to matrix interference impose stringent requirements on detection sensitivity and specificity [107]. Composite Raman probes, benefiting from ultrahigh signal-to-noise ratios (SNRs), programmable signal amplification strategies, and excellent anti-interference capability, have demonstrated clear advantages in nucleic acid analysis.

In miRNA detection, Zhu et al. achieved disordered amplification and ultrasensitive detection of the cancer-related gene miR-21, providing a new strategy for low-abundance miRNA analysis [100]. Chen et al. further demonstrated reliable discrimination of miR-21 at different concentrations in simulated serum environments corresponding to various cervical cancer lesion grades [97]. For the liver cancer biomarker miR-222, Man S. et al. realized rapid detection over a wide dynamic range (1 fM–10 nM) and successfully applied the method to liver cancer cell lysates and human serum samples [86].

For ctDNA and mutation analysis, Liang et al. achieved high-sensitivity ctDNA detection in complex serum matrices with good linearity across five orders of magnitude [88]. Wang et al. reported ultrasensitive detection of the liver cancer-related mutant PIK3CA E542K [84], while Chen et al. achieved highly sensitive detection of the tumor suppressor gene P53, with cross-validation ensuring analytical accuracy [92].

High-specificity recognition of viral DNA and tumor subtype–related sequences has also been extensively explored. Ye et al. achieved sensitive and selective detection of cervical cancer–associated HPV-16 and HPV-18 DNA, effectively discriminating these subtypes from other HPV variants in clinical samples [96]. Similarly, Liu et al. reported ultrasensitive and highly specific detection of HPV-58 DNA, maintaining excellent anti-interference performance in complex human serum [87].

Overall, composite Raman probes have demonstrated significant advantages in ultra-low detection limits, wide dynamic range, and adaptability to complex clinical samples in the detection of tumor nucleic acid markers, providing strong technical support for early screening, molecular typing, and personalized treatment monitoring of tumors.

#### 3.1.2. Protein and Receptor Biomarkers

Protein and receptor biomarkers provide critical molecular insights into tumor development, progression, and therapeutic response. Owing to their multiplex encoding capacity, high SNR, and robustness against biological background signals, composite Raman probes have achieved significant progress in protein and receptor detection across diverse tumor models.

At the cellular level, Bagheri et al. constructed multichannel SERS probes capable of simultaneously detecting human epidermal growth factor receptor 2 (HER2), estrogen receptor (ER), and progesterone receptor (PR) in breast cancer cell lines with high specificity and sensitivity. By employing different Raman reporters, four-channel synchronous multiplex imaging was achieved, enabling accurate identification of low-abundance target cells within a high-background negative cell population [29]. Li et al. demonstrated simultaneous ultrasensitive detection of bladder cancer biomarkers FGFR and NMP22, highlighting the advantage of SERS probes in multivariate biomarker analysis [49]. Cheng et al. employed a ratiometric Raman probe to detect hCE1 in HepG-2 liver cancer cells, further illustrating the utility of ratio-based signal output in intracellular sensing [103].

Beyond cell models, composite Raman probes have been successfully applied to complex biological samples, particularly serum. Wang et al. achieved sensitive and specific detection of prostate cancer biomarker PSA and gastric cancer marker CD44 [94]. Suleimenova et al. employed a MIP-functionalized paper substrate integrated with silver nanostars to establish a sandwich-type SERS assay for the sensitive detection of nucleolin (NCL), a cancer-related multifunctional protein, in complex human serum [65]. Zhuang et al. reported rapid and sensitive detection of gastric cancer-related proteins matrix metalloproteinase-9 (MMP-9) and IL-6 in serum [98], while Sun et al. achieved accurate quantitative analysis of MMP-9 [51]. Lan et al. realized highly sensitive and specific quantification of PSA [24], and Yanling Wang developed a test-strip-based strategy for ultrasensitive detection of the kinase biomarker PEAK1 in cell lysates and plasma [20].

Additional studies further demonstrate the clinical relevance of composite Raman probes. Zheng et al. achieved precise quantification of total PSA in serum, enabling reliable discrimination within the clinical diagnostic gray zone [21]. Xia et al. combined SERS detection of circulating glycoprotein MUC1 with machine learning to accurately distinguish breast cancer patients at different stages from healthy individuals [47]. Qiu et al. differentiated bladder cancer patients by monitoring hyaluronidase activity in urine samples [93], while Yao et al. quantified chiral metabolites D-proline and D-alanine in the saliva of gastric cancer patients [99]. At the single-molecule level, Sharma et al. achieved SERS detection of EGFR by correlating Raman signals with atomic force microscopy (AFM) characterization [41]. In clinical sample analysis, Hwang et al. [56] and Hao et al. [54] both demonstrated quantitative detection of the pancreatic cancer marker CA19-9.

#### 3.1.3. Exosomes

Exosomes, also referred to as extracellular vesicles (EVs), are actively secreted by tumor cells and carry rich molecular information, including proteins, nucleic acids, and lipids. Their close association with tumor status makes them highly promising biomarkers for early diagnosis, tumor typing, and therapeutic monitoring. However, their small size, high heterogeneity, and coexistence with abundant background vesicles pose substantial challenges for sensitive and multiplexed detection [108].

Composite Raman probes provide effective solutions for precise EV analysis. For endogenous EV biomarker detection, He et al. successfully distinguished gastric cancer patients from healthy controls by monitoring tumor-derived exosomal miRNAs in clinical serum samples [85]. Diao et al. differentiated cervical cancer patients by detecting surface CD63 protein variations on exosomes [91]. Bashir et al. achieved ultrasensitive detection of placental alkaline phosphatase–positive exosomes in ovarian cancer patient plasma [55]. In related work, tumor-derived exosomes from ovarian and breast cancer patient serum were specifically captured using CD9 antibody functionalization, followed by catalytic oxidation of *o*-phenylenediamine to generate characteristic SERS signals, enabling reliable discrimination between cancer patients and healthy individuals [76]. Ho et al. developed a SERS based droplet microfluidic platform to rapidly detect HER2 positive exosomes of breast cancer through salt induced aggregation of AuNPs on the tablet [106].

Beyond single-marker detection, composite Raman probes have demonstrated strong capability in EV heterogeneity analysis and multiplex profiling. Xia et al. detected EpCAM-positive MCF-7 cells at concentrations as low as 3 cells mL^−1^ in whole blood without enrichment and effectively distinguished breast cancer patient–derived exosomes from healthy controls [39]. Ngo et al. demonstrated sensitive detection of cancer-derived small EVs in complex plasma matrices [23]. Chen et al. integrated SERS analysis with deep learning algorithms to achieve high-resolution molecular heterogeneity analysis and subtype classification of non-small cell lung cancer exosomes [25].

At the single-cell and multiplex level, Ahmed et al. reported multiplex detection of four immune checkpoint proteins on EVs derived from HCC827 lung cancer cells and dynamically monitored expression changes during EGFR inhibitor treatment [69]. In another study, high-sensitivity multiplex detection of three ovarian cancer EV surface proteins enabled effective differentiation among healthy, benign, and malignant clinical samples [57].

### 3.2. Tissue-Level Tumor Detection

Tissue-level detection centers on the tumor mass and its associated microenvironment, capturing structural features, functional states, and dynamic changes at the histological scale. By leveraging Raman imaging and advanced signal analysis, this approach yields multidimensional macroscopic information beyond molecular-level assays, supporting tumor diagnostic profiling, microenvironmental assessment, treatment guidance, and therapeutic monitoring.

#### 3.2.1. Imaging-Based Tumor Diagnosis

Bioimaging plays a central role in tumor diagnosis, staging, image-guided biopsy, and metastasis surveillance. Conventional imaging modalities, such as MRI, Computed Tomography (CT) and PET, primarily provide macroscopic anatomical or functional information but are often limited in molecular specificity. In contrast, Raman imaging enables real-time mapping of molecular vibrational fingerprints at cellular and subcellular resolutions [109], establishing a foundation for imaging-based diagnosis, tumor subtyping, and precise intraoperative localization.

**Figure 10:**
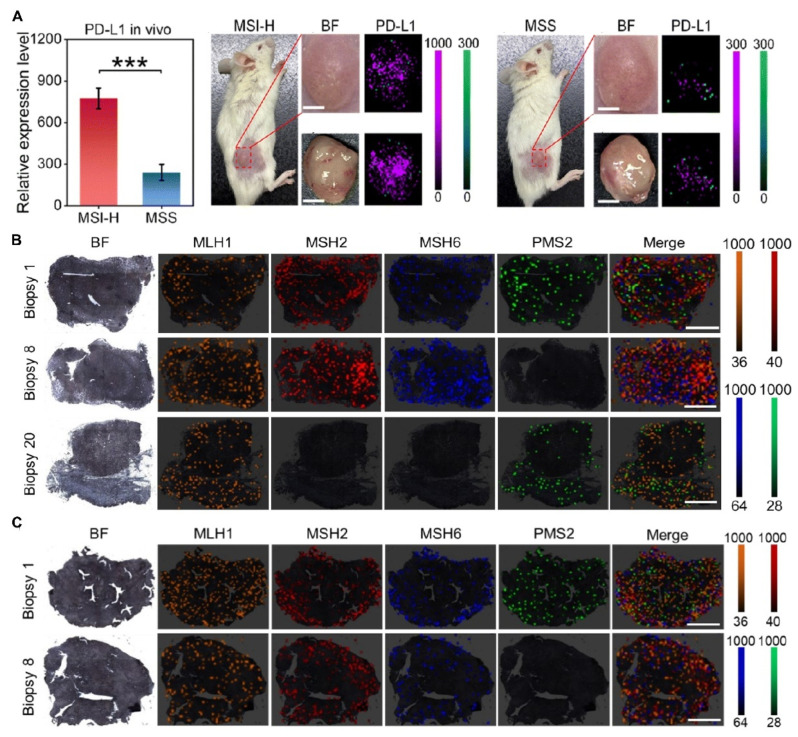
(**A**) Detection of PD-L1 protein in CRC mice with PDX MSI-H and PDX MSS, in vitro Raman imaging of PD-L1 protein in tumors. (**B**) Raman imaging of MMR proteins in three representative gastric cancer biopsies. (**C**) Raman imaging of MMR proteins in two representative breast cancer biopsies. Reproduced from [42], Royal Society of Chemistry, 2025.

In the context of tumor subtyping and metastasis monitoring, Fu et al. developed a multicolor O-GERTs-based Raman probe capable of simultaneously detecting four mismatch repair (MMR) proteins in cancer tissues, enabling multiplexed assessment of microsatellite instability-related molecular features. As shown in Figure 10A, reliable discrimination between microsatellite-stable (MSS) and microsatellite high-frequency (MSI-H) colorectal cancer (CRC) subtypes was achieved through in vitro Raman imaging of PD-L1 expression in a patient-derived xenograft (PDX) CRC mouse model. In addition, Figure 10B,C present representative Raman images of four MMR proteins in gastric cancer and breast cancer tissues, respectively, further demonstrating the applicability of this platform across different tumor types [42]. Zhang et al. enabled real-time monitoring of micrometastases in an in vivo triple-negative breast cancer model by integrating SERS and bioluminescence imaging, allowing comprehensive evaluation of tumor growth and therapeutic response [43]. Tanwar reported rapid and selective imaging of metastatic prostate cancer cells characterized by high expression of legumain [90]. For tumor-specific imaging, Liu et al. achieved high-contrast SERS imaging combined with dark-field microscopy in T24 bladder cancer cells [53], while Cheng et al. demonstrated effective differentiation of bladder cancer cells via in vitro SERS imaging and further tracked tumor progression in ex vivo samples [32]. In addition, Wu et al. performed targeted SERS imaging of DU145 cells, successfully distinguishing prostate cancer cells with high versus low metastatic potential [40].

#### 3.2.2. Tumor Microenvironment Analysis and Metabolism

The TME and its associated metabolic reprogramming play pivotal roles in tumorigenesis, invasion, and therapeutic response. Unlike single molecular biomarkers, the TME is spatiotemporally dynamic and highly heterogeneous, necessitating detection technologies with high spatial resolution, rapid temporal responsiveness, and strong resistance to background interference [110]. Composite Raman probes, characterized by their spatiotemporal resolution and anti-interference capability, offer powerful tools for precise TME characterization and metabolic analysis.

**Figure 11:**
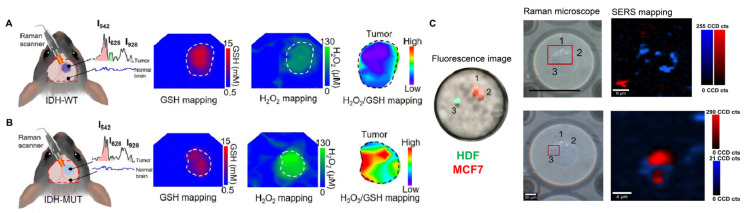
(**A**) Distribution map of GSH and H_2_O_2_ exposed tumor areas in the IDH1-WT glioma model and (**B**) IDH1-MUT glioma model. Reproduced from [101], Wiley-VCH GmbH, Weinheim, 2025. (**C**) Transfer process of MCF-7 EV from MCF-7 to HDF. Reproduced from [22], Wiley-VCH GmbH, Weinheim, 2025.

In the detection of key TME factors, Yu et al. achieved high-sensitivity, cross-scale analysis of matrix metalloproteinase activity within the medulloblastoma microenvironment, accurately distinguishing patients from healthy controls in clinical serum samples [95]. For dynamic TME monitoring and functional analysis, Dey et al. realized non-invasive, dynamic pH monitoring across various 3D tumor spheroids [102]. Integrating deep learning, Yin et al. generated intraoperative metabolic distribution maps of fresh tumor tissues, enabling the genotyping of IDH1 wild-type (IDH1-WT) in Figure 11A versus IDH1 mutant (IDH1-MUT) gliomas in Figure 11B with high classification accuracy in 31 patient samples [101]. Another study employed SERS imaging to monitor the real-time, in situ intercellular communication mediated by EVs between MCF-7 breast cancer cells and human dermal fibroblasts (HDFs). As shown in Figure 11C, the dynamic transfer of EVs from MCF-7 cells to HDFs was tracked over a four-day period, demonstrating the capability of SERS imaging for longitudinal visualization of EV-mediated cellular interactions [22]. Additionally, Wang exploited the differences in electron transfer capabilities between cancerous and normal cells to achieve specific recognition and the dynamic monitoring of apoptosis [74].

#### 3.2.3. Tumor Therapy and Efficacy Monitoring

With the continuous advancement of composite Raman probes in imaging and analysis, their functional scope has expanded from passive detection toward integrated theranostic platforms. These probes offer distinct advantages in guiding tumor treatment, clearing residual lesions, and enabling intraoperative and longitudinal efficacy monitoring.

**Figure 12:**
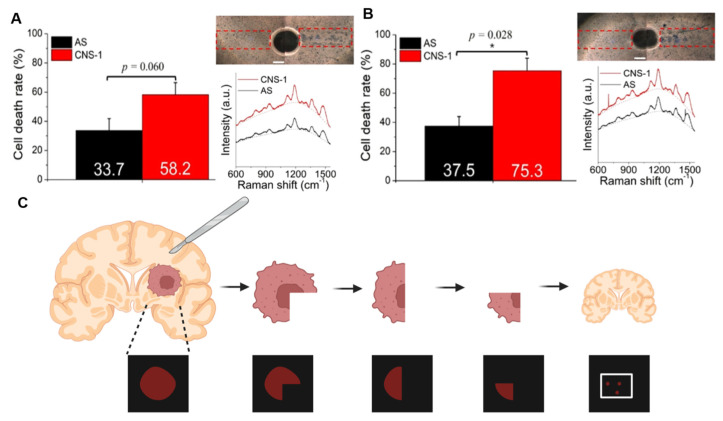
CNS-1 and AS exhibit Raman contrast and selective photothermal elimination under (**A**) 1 min and (**B**) 3 min photothermal irradiation. Reproduced from [50], American Chemical Society, 2025. (**C**) Schematic diagram of surgical resection steps in Raman guided surgery.

In image-guided therapy, Mellor et al. designed Raman probes that inhibited tumor growth in mouse models through photothermal effects combined with intratumoral injection and laser irradiation [52]. Guo et al. achieved high-sensitivity tumor imaging and real-time monitoring, triggering photothermal therapy under NIR irradiation while simultaneously activating an oxygen-independent photosensitizer to suppress tumor progression [31]. Chang et al. demonstrated Raman contrast-guided selective photothermal ablation in a coculture system of glioblastoma (GBM) cells (CNS-1) and normal astrocytes (AS). As shown in Figure 12A,B, effective Raman contrast and tumor-selective photothermal elimination were achieved under 1 min and 3 min laser irradiation, respectively, highlighting the capability of Raman-guided photothermal therapy for selective cancer cell targeting [50]. Kircher et al. further showed that Raman imaging could clearly delineate tumor margins and identify microscopic infiltrations invisible to the naked eye, successfully guiding intraoperative tumor resection [6]. As shown in pattern diagram 12C, the tumor was sequentially resected in a stepwise manner, with intraoperative Raman imaging performed after each partial removal until no residual tumor was apparent by visual inspection. Notably, following gross tumor resection, several discrete Raman signal foci were detected within the resection bed. Histological analysis confirmed tumor infiltration at these sites, revealing finger-like extensions of tumor tissue into the surrounding brain parenchyma. Subsequent studies have integrated Raman image-guided surgery with photothermal ablation of residual lesions, while simultaneously inducing immune memory to reduce recurrence risk [80]. Furthermore, Zhang et al. achieved ultra-sensitive detection and imaging of APE1 and miR-155 in breast cancer cells, followed by targeted ablation under 808 nm laser irradiation [89]. Song et al. demonstrated rapid NIR-II handheld Raman scanner-guided resection of 4T1 breast tumors within 8 min [30].

Beyond therapeutic intervention, composite Raman probes have also enabled treatment evaluation and prognosis assessment. Liu et al. systematically evaluated probe targeting efficiency across 2D cells, 3D spheroids, and in vivo tumor models, guiding the rational design of tumor-targeted delivery systems [44]. Fan et al. enabled multiplex detection of surface markers on lung cancer CTCs and combined with algorithmic analysis, differentiated metastatic from non-metastatic patients while capturing phenotypic evolution during longitudinal monitoring to inform therapeutic decision-making [48].

## 4. Challenges and Bottlenecks

**Figure 13:**
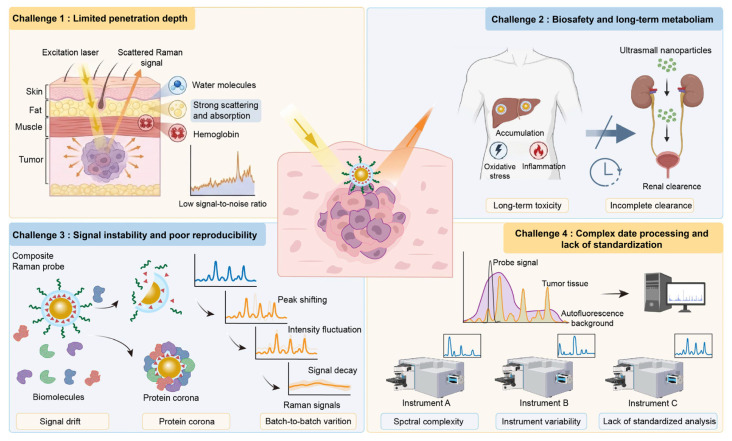
Challenges faced by composite Raman probes in oncology.

While composite Raman probes exhibit promising potential in precision tumor diagnosis, intraoperative navigation, and therapeutic evaluation, substantial challenges remain in bridging fundamental research and clinical translation. Critical issues persist across materials design, synthesis reproducibility, in vivo biological behavior, biosafety evaluation, and data analysis, as shown in Figure 13. These challenges are systematically categorized and discussed in the following four sections.

### 4.1. Optical Penetration Limits in Deep-Tissue Imaging

Raman spectroscopy relies on the inelastic scattering of photons by molecular bonds. Nevertheless, the strong absorption and scattering properties of biological tissues severely restrict optical penetration, limiting the applicability of composite Raman probes for deep-seated tumor diagnosis. Major tissue components, including hemoglobin, water, and lipids, exhibit strong absorption in the visible and near-infrared regions. Combined with the intrinsically weak nature of Raman scattering, this ultimately leads to an extremely low SNR for signals originating from deep tissues.

For composite Raman probes, both excitation delivery and signal collection are constrained by tissue penetration depth. When targeting deep-seated solid tumors, excitation light undergoes substantial absorption and scattering before reaching the lesion, markedly reducing excitation efficiency. At the same time, Raman signals generated within the tumor are further attenuated during outward propagation, rendering the detected signal comparable to or indistinguishable from background noise. Moreover, tumor heterogeneity, which includes irregular vascular distribution and variable fibrosis, induces stochastic variations in photon propagation paths, further exacerbating signal attenuation and distortion. Although strategies including NIR-II excitation and photoacoustic-Raman multimodal imaging have partially alleviated these limitations, Zhang et al. achieved non-invasive imaging of simulated lesions in mice with a thickness of 1.5 cm by combining ultra bright SERS nanotags and transmission Raman spectroscopy technology, which is a breakthrough in traditional technology limitations [111]. Therefore, reliable non-invasive imaging of tumors located deeper than 1 cm remains limited, representing a critical bottleneck for the clinical translation of composite Raman probes.

### 4.2. Biosafety and Long-Term Metabolic Fate

Biosafety is a fundamental prerequisite for clinical translation. The structural complexity and compositional diversity of composite Raman probes complicate the evaluation of their in vivo metabolism and long-term safety. While core components such as noble metal nanoparticles and carbon-based materials offer superior optical properties, they may pose potential biosafety risks. Small-sized noble metal nanoparticles can internalize into cells, disrupting mitochondrial function and inducing oxidative stress or DNA damage [112,113], Carbon-based materials are prone to accumulation in the liver and spleen, potentially triggering chronic inflammation and tissue fibrosis [114]. In addition, heavy metal ion leakage from quantum dots may induce long-term toxic effects [115,116]. These risks are further amplified when probe materials exhibit poor biodegradability or release toxic degradation products.

The long-term metabolic fate and clearance of composite Raman probes constitute another major concern. Owing to wide variations in size, morphology, surface charge, and chemical composition, these probes exhibit diverse metabolic pathways and clearance profiles. Many probes, particularly those with large hydrodynamic diameters or high hydrophobicity, cannot be efficiently eliminated through renal filtration. Their prolonged retention within the reticuloendothelial system (RES) may induce chronic inflammation and organ damage [117]. At present, systematic studies addressing long-term toxicity, pharmacokinetics, and biocompatibility remain limited, especially in clinically relevant tumor models.

Future research should prioritize biocompatible material design without compromising Raman performance. Elucidating in vivo metabolic pathways and clearance mechanisms, together with establishing rigorous biosafety evaluation frameworks, will be essential for advancing the clinical application of composite Raman probes.

### 4.3. Signal Stability and Reproducibility

Signal stability and reproducibility are fundamental prerequisites for precise tumor detection and remain major obstacles to the widespread application of composite Raman probes.

From a materials perspective, composite Raman probes typically integrate Raman-enhancing substrates (e.g., noble metal nanostructures, carbon materials, or semiconductors) with functional carriers such as polymers or biomacromolecules. Interfacial instability may result in structural collapse, aggregation, or degradation under shear stress in biological fluids or enzymatic attack, leading to signal attenuation or fluctuation [118]. Furthermore, variations in particle size distribution, morphology, and surface modification strongly influence enhancement efficiency. The lack of standardized synthesis protocols often leads to batch-to-batch variability, undermining diagnostic reproducibility.

From a biological perspective, the TME, characterized by acidity, hypoxia, and elevated ROS, may chemically interact with composite materials, compromising probe integrity. In addition, nonspecific adsorption of biomolecules leads to protein corona formation, altering surface charge and optical properties and potentially shifting or obscuring characteristic Raman peaks [119,120]. During in vivo imaging, non-uniform probe distribution, hemodynamic fluctuations, and tumor heterogeneity further introduce spatiotemporal variability in signal intensity.

Accordingly, improving structural robustness, minimizing nonspecific adsorption, and establishing standardized signal acquisition and analysis protocols are critical for enhancing the reliability and reproducibility of composite Raman probes.

### 4.4. Complexity in Data Processing and Lack of Standardization

Raman spectra acquired from tumor tissues represent complex superpositions of signals from diverse biomolecular components, resulting in highly convoluted spectral profiles. Endogenous tissue autofluorescence and fluorescence originating from probe carrier materials often exceed Raman signal intensity, yielding spectra with extremely low SNRs that are difficult to interpret. In addition, spatial heterogeneity and temporal dynamics are inherent to in vivo imaging, which further complicates spectral analysis.

Beyond biological interference, Raman spectroscopy and imaging generate large-scale datasets. Disease subtype discrimination based on subtle spectral differences requires the analysis of vast numbers of spectra, imposing high demands on computational resources and analytical algorithms. Inter-instrument variability further exacerbates this challenge. Differences in laser wavelength, spectral resolution, acquisition parameters, and detector sensitivity across Raman systems result in deviations in peak position, intensity, and line shape. Even within identical instrument models, factors such as optical alignment and laser stability may introduce inconsistencies.

Regarding standardization, there is currently no unified workflow for Raman spectral data processing. Preprocessing methods vary significantly across research teams, leading to divergent results for identical samples. Secondly, there is a lack of unified metrics for evaluating algorithm performance, making it difficult to objectively assess different processing methods. Thirdly, clinical detection demands rapid, accurate, and stable results, whereas current processing of complex data consumes excessive computational resources and time. Consequently, developing efficient, reliable data analysis models combined with machine learning is an indispensable pathway to enhancing data credibility and driving clinical implementation.

## 5. Conclusions and Perspectives

Emerging at the intersection of materials science, optical spectroscopy, and oncology, composite Raman probes have demonstrated unique advantages in precision tumor diagnosis and therapy. This review has systematically delineated the design principles, construction strategies, oncological applications, and current bottlenecks of these probes, synthesizing representative advancements from the past three years into a coherent conceptual framework.

Regarding design principles, signal amplification in composite Raman probes arises from the synergistic coupling of electromagnetic enhancement, chemical enhancement, and optical field modulation. Noble metal nanostructures provide the foundation for high-efficiency signal amplification through LSPR, while the integration of Raman reporters, biorecognition units, and functional carriers imparts biocompatibility, targeting specificity, and environmental responsiveness. This shift marks a transition from single-mode signal amplification to multifunctional probe integration. In terms of construction strategies, plasmonic engineering, interfacial electron modulation, stimuli-responsive dynamic regulation, and intrinsic molecular scattering enhancement each have their own advantages. These strategies optimize nanostructure morphology, regulate interfacial charge transfer, respond to TME signals, or enhance intrinsic scattering properties to address diverse diagnostic and therapeutic requirements.

In biomedical applications related to oncology, composite Raman probes have, at the research level, established a relatively systematic application framework encompassing molecular imaging, biomarker detection, TME analysis, and integrated theranostic strategies. In biomarker detection, these probes have enabled ultrasensitive and highly specific analysis of key biomolecules, which include nucleic acids, proteins, and exosomes, providing strong technical support for early tumor screening. In the imaging domain, the combination of high spatial resolution and intrinsic molecular fingerprinting allows accurate tumor subtyping, metastasis monitoring, and intraoperative navigation. Furthermore, in TME analysis and therapy-related applications, real-time monitoring of microenvironmental factors such as pH and ROS, together with therapeutic feedback, has further advanced the development of precision oncology. Nevertheless, challenges related to signal stability, biosafety, deep-tissue imaging capability, and data-processing standardization remain unresolved. These issues collectively impede the translation of composite Raman probes from fundamental research toward routine clinical application.

Although significant progress has been made in the use of composite Raman probes for tumor treatment and diagnosis, there are still several key knowledge gaps in current research that have not been resolved. Firstly, the quantitative synergistic mechanism between EM, CM and intrinsic molecular Raman enhancement has not been fully elucidated. Most studies focus on a single enhancement pathway rather than their combined contributions, which limits rational probe design. Secondly, there are currently no composite Raman probes in clinical trials, and the detection and treatment of tumors are limited to the collection of animal or clinical samples to validate the capabilities of a certain probe system. The hidden problems in these research gaps also indicate the direction we should strive for.

Looking ahead, the future development of composite Raman probes is expected to focus on three major directions: performance optimization, functional integration, and clinical translation, driven by deep interdisciplinary convergence among materials science, medicine, and information science.

In terms of material design and performance optimization, improving nanostructural uniformity, structural stability, and controllable in vivo metabolic behavior will represent key breakthroughs. Although composite Raman probes combined with microfluidic platforms and template-assisted substrates have enabled reliable in vitro detection, there remains a pressing need to develop carrier materials that simultaneously exhibit excellent biocompatibility and biodegradability, such as biodegradable polymers and biomineralized nanoparticles. In parallel, advanced surface functionalization strategies should be implemented to mitigate protein corona formation and nonspecific adsorption, thereby improving biosafety and signal stability. Moreover, the integration of NIR-II excitation with multimodal imaging techniques, which include Raman-photoacoustic and Raman-MRI imaging, is expected to further enhance deep-tissue detection capability and overcome current limitations in the non-invasive imaging of large solid tumors.

With respect to functional integration, multimodal synergy and intelligent responsiveness will constitute major development trends. On one hand, combining Raman imaging with therapeutic modalities such as photothermal therapy and immunomodulation enables the construction of multifunctional probes that integrate diagnosis, therapy, and monitoring into a single platform, thereby improving treatment precision and efficiency. On the other hand, the development of intelligent probes capable of synergistically responding to multiple tumor microenvironmental cues will facilitate dynamic monitoring of tumor progression and provide real-time guidance for personalized therapeutic adjustment. Meanwhile, multiplexed detection systems based on bioorthogonal Raman probes are expected to further enhance simultaneous multi-biomarker analysis, improving the accuracy of tumor subtyping and early diagnosis.

From the perspective of clinical translation, there is an urgent need to establish standardized probe performance evaluation criteria and unified data processing workflows. The integration of efficient machine learning algorithms to streamline spectral analysis, together with the development of miniaturized and automated Raman detection devices, will be essential to meet clinical application requirements. In addition, large-scale, multi-center clinical studies are required to systematically validate the diagnostic efficacy and biosafety of composite Raman probes across diverse tumor types, thereby providing robust evidence-based support for their clinical adoption.

In summary, composite Raman probes hold substantial promise in oncological diagnosis and therapy. Although significant challenges remain, continued advances in materials synthesis, spectroscopic techniques, and artificial intelligence are expected to accelerate their translational progress. Ultimately, these probes are poised to play an increasingly important role in early cancer detection, precision treatment, and prognostic evaluation, providing strong technological support for the long-term goal of achieving earlier detection, earlier diagnosis, and earlier treatment of cancer.

## Figures and Tables

**Table 1:** Comparison of key parameters for detecting different tumor related biomolecule markers using different composite Raman probes.

Biomarker Category	Target Analyte	Cancer Type	Probe Type	EF	LOD	Recovery	Study
Nucleic acids	miR-21	Cervical cancer	thiol-modified DNA probes and AgNPs	/	1.28 × 10^−6^ μmol/L	/	[97]
	miR-21	Cervical cancer	Raman labeling based on AuNPs combined with lateral flow assay	/	8.96 aM	92.0–100.8%	[100]
	miR-222	Liver cancer	hot spot aggregation based on gold nanospheres and DCHA signal amplification	/	0.33 fM	97.19–102.51%	[86]
	miR-1246/ miR-106a	Prostate cancer/ Gastric cancer	double surface glass micropipette coated with AuNPs	/	1 aM	/	[94]
	ctDNA	/	ordered assembly of DNA modified AuNP Raman tags mediated by Mg^2+^	/	10.8 fmol/L	91.33–100.40%	[88]
	PIK3CA E542K	Liver cancer	CHA reaction combined with Au@Pt SERS technology and nanoenzyme binding	/	4.12 aM	/	[84]
	P53 Gene	/	AuNPs-COF nanostructure combined with HCR for signal amplification	/	6.70 pM	93.0–105%	[92]
	HPV16/18	Cervical cancer	Au@Ag SERS tag combined with CRISPR/Cas12a system	/	HPV16: 0.347 fM HPV18: 0.738 fM	/	[96]
	HPV58	Cervical cancer	Au hexagonal plate and PER based DNA cube captor	/	5.12 fM	95.52–103.10%	[87]
Proteins/ Receptors	HER2/ER/PR	Breast cancer	multiplexed detection based on AuNPs	/	/	/	[29]
	FGFR3/NMP22	Bladder cancer	AgAu@Ag core shell triangular nanonet combination SERS substrates	1.88 × 10^8^	FGFR3: 73.36 fg/mL NMP22: 21.56 fg/mL	FGFR3:99.2–107.5% NMP22: 98.9–104.8%	[49]
	hCE1	Liver cancer	ratio type probe based on Ag Au Nanoflowers	2.1 × 10^9^	7.3 pg/mL	/	[103]
	PSA/ CD44	Prostate cancer/ Gastric cancer	double surface glass micropipette coated with AuNPs	/	0.001 ng/mL	/	[94]
	PSA	Prostate cancer	AuNRs probe combined with polymer AgNCs substrate	/	5.6 × 10^−10^ mg/mL	/	[24]
	t-PSA	Prostate cancer	Ag-Au nanoflowers combined with magnetic enrichment	/	100 fg/mL	91.0–110.3%	[21]
	NLC	/	Ag nanostars combined with MIP substrate	/	0.068 nmol/L	/	[65]
	MMP-9/ IL-6	Gastric cancer	Au nanobipyramids with Ag shells combined with Au nano-hexagonal arrays chip	Au@Ag: 3.71 × 10^6^ Au chip: 2.27 × 10^8^	MMP-9: 0.263 pg/mL IL-6: 0.195 pg/mL	/	[98]
	MMP-9	/	the shell core satellite structure of Au combined with lateral flow immunoassays	/	0.051 ng/mL	86.40–98.22%	[51]
	PEAK1	/	Ag nanoflowers combined with lateral flow immune assays	2.8 × 10^8^	1 fg/mL	98.4–99.6%	[20]
	MUC1	Breast cancer	Au-Ag core–shell NPs combined with magnetic nanobeads	4.20 × 10^6^	2.96 fg/mL	/	[47]
	HAase	Bladder cancer	hyaluronic acid-coated AgNPs	/	3 × 10^−4^ U/mL	/	[93]
	CA19-9	Pancreatic cancer	SiO_2_ core and Au-Ag Janus satellite	3.79 × 10^8^	3.67 × 10^−5^ IU/mL	96.4–101.7%	[54]
	CA19-9	Pancreatic cancer	confined growth of Ag nanogap shells inside mesoporous Si NPs	1.5 × 10^6^	0.025 U/mL	86.5–104.8%	[56]
	EGFR	/	DNA origami and AuNR dimer nanoantennas	/	0.2 nM	/	[41]
	D-Pro/ D-Ala	Gastric cancer	polyphenol briged Co-Ag bimetallic heterostructure	/	D-Pro: 2.37 μM D-Ala: 2.15 μM	98.6–109.4%	[99]
Exosomes	/	Gastric cancer	AuNPs tag combined with CRISPR/Cas13a and AgNRs@DNA tetrahedron probes	/	6.1 × 10^3^ particles/mL	94.87–108.44%	[85]
	/	Cervical cancer	AuNPs nanomembrane combined with AuNP bio-barcode	/	4.7 × 10^5^ particles/mL	81.5–106.2%	[91]
	/	Ovarian cancer	AuNPs combined with magnetic Beads	/	1.5 × 10^5^ particles/μL	/	[55]
	/	Ovarian cancer	MOF-derived iron oxide nanorods and SERS barcode based on mesoporous AuNPs	/	2.13 EVs/μL	/	[57]
	/	Ovarian cancer/ Breast cancer	Ag@lacunary polyoxometalates nanoclusters	/	7.73 × 10^5^ particles/mL	/	[76]
	/	Breast cancer	assemble AuNPs in triangular pyramid DNA	/	1.1 × 10^2^ particles/μL	/	[39]
	/	Breast cancer	aggregation of AuNPs in droplet microfluidic platform	/	4.5 log_10_ particles/mL	/	[106]
	/	Lung cancer	capture unit based on AuNCs and microfluidic technology	/	BEAS-2B: 1.64 × 10^6^ particles/mL H460: 2.66 × 10^5^ particles/mL H226: 5.08 × 10^5^ particles/mL PC-9: 2.64 × 10^5^ particles/mL	/	[25]
	/	Lung cancer	AuNPs SERS tags combined with mesoporous Au chips	/	/	/	[69]

## Data Availability

No new data were created or analyzed in this study.

## Abbreviations

The following abbreviations are used in this manuscript:

SERS	surface-enhanced Raman scattering
EM	electromagnetic mechanism
CM	chemical mechanism
CTC	circulating tumor cell
NIR	near-infrared
PTT	photothermal therapy
PDT	photodynamic therapy
TME	tumor microenvironment
ROS	reactive oxygen species
LSPR	localized surface plasmon resonance
EF	enhancement factor
AuNR	gold nanorod
AuNC	gold nanocube
TEM	transmission electron microscope
BF-STEM	bright-field scanning transmission electron microscopy
PSMA	poly (styrene-maleic acid)
E_EM_	electromagnetic enhancement factor
NP	nanoparticle
CJS	core-janus-satellite
MIP	molecularly imprinted polymers
BSA	bovine serum albumin
CT	charge transfer
NDC	nitrogen-doped carbon
NC	nanocage
DOS	density of states
POM	polyoxometalate
HOMO	highest occupied molecular orbital
LUMO	lowest unoccupied molecular orbital
ELF	electron localization function
TMD	transition metal dichalcogenide
MOF	metal–organic framework
D-A	donor-acceptor
PET	positron emission tomography
SICTERS	stacking-induced charge-transfer-enhanced Raman scattering
SRS	stimulated Raman scattering
COF	covalent organic framework
SHINERS	shell-isolated nanoparticle-enhanced Raman spectroscopy
ctDNA	circulating tumor DNA
CHA	catalytic hairpin assembly
TMB	3,3′, 5,5′—tetramethylbenzidine
miRNA	microRNA
DCHA	dual-catalyst hairpin assembly
PER	primer exchange reaction
HCR	hybridization chain reaction
TA	tannic acid
3-MPBA	3-mercaptophenylboronic acid
4-MBA	4-mercaptobenzoic acid
SNR	signal-to-noise ratio
HER2	human epidermal growth factor receptor 2
PIK3CA E542K	phosphatidylinositol-4,5-bisphosphate 3-kinase catalytic subunit alpha E542K mutant
ER	estrogen receptor
PR	progesterone receptor
FGFR	fibroblast growth factor receptor
NMP22	nuclear matrix protein 22
hCE1	human carboxylesterase 1
PSA	prostate specific antigen
LOD	limit of detection
NCL	nucleolin
MMP-9	matrix metalloproteinase-9
IL-6	interleukin-6
PEAK1	pseudopodium-enriched atypical kinase 1
MUC1	mucin 1
EGFR	epidermal growth factor receptor
AFM	atomic force microscopy
CA19-9	carbohydrate antigen 19-9
EV	extracellular vesicle
EpCAM	epithelial cell adhesion molecule
MCF-7	michigan cancer foundation-7
MPP	micropapillary pattern
MRI	Magnetic Resonance Imaging
CT	Computed Tomography
MMR	mismatch repair
MSS	microsatellite-stable
MSI-H	microsatellite high-frequency
CRC	colorectal cancer
PDX	patient-derived xenograft
PD-L1	programmed cell death ligand 1
HDF	human dermal fibroblast
GBM	glioblastoma
AS	astrocytes
APE1	apurinic-apyrimidinic endonuclease 1
RES	reticuloendothelial system
